# Silicon mitigates nutritional stress in quinoa (*Chenopodium quinoa* Willd.)

**DOI:** 10.1038/s41598-021-94287-1

**Published:** 2021-07-19

**Authors:** Ana Carolina Sales, Cid Naudi Silva Campos, Jonas Pereira de Souza Junior, Dalila Lopes da Silva, Kamilla Silva Oliveira, Renato de Mello Prado, Larissa Pereira Ribeiro Teodoro, Paulo Eduardo Teodoro

**Affiliations:** 1 Universidade Federal do Mato Grosso do Sul, Campus de Chapadão do Sul – UFMS/CPCS, Chapadão do Sul, MS Brazil; 2 Faculdade de Ciências Agrárias e Veterinárias – UNESP/FCAV, Universidade Estadual Paulista “Júlio de Mesquita Filho”, Jaboticabal, SP Brazil

**Keywords:** Plant physiology, Plant stress responses

## Abstract

Nutritional deficiency is common in several regions of quinoa cultivation. Silicon (Si) can attenuate the stress caused by nutritional deficiency, but studies on the effects of Si supply on quinoa plants are still scarce. Given this scenario, our objective was to evaluate the symptoms in terms of tissue, physiological and nutritional effects of quinoa plants submitted to nitrogen (N), phosphorus (P), potassium (K), calcium (Ca), and magnesium (Mg) deficiencies under Si presence. The experiment consisted of a factorial scheme 6 × 2, using a complete solution (CS), -N, -P, -K, -Ca, -Mg combined with absence and presence of Si (1.5 mmol L^−1^). Symptomatic, physiological, nutritional and evaluation vegetative were performed in quinoa crop. The deficiencies of N, P, K, Ca and Mg in quinoa cultivation caused visual symptoms characteristic of the deficiency caused by respective nutrients, hence decreasing the plant dry mass. However, Si supply attenuated the deficiency effects by preserving the photosynthetic apparatus, increasing the chlorophyll production, increasing the membrane integrity, and decreasing the electrolyte leakage. Thus, the Si supply attenuated the visual effects provided by deficiency of all nutrients, but stood out for N and Ca, because it reflected in a higher dry mass production. This occurred because, the Si promoted higher synthesis and protection of chlorophylls, and lower electrolyte leakage under Ca restriction, as well as decreased electrolyte leakage under N restriction.

## Introduction

Quinoa (*Chenopodium quinoa* Willd.) is a valuable food source due to its high nutritional potential^1^. Its seeds have high quality proteins, vitamins, and essential amino acids, including lysine, methionine, and threonine, which are lacking in the seeds of legumes and cereals^2–4^.

The main cause of income losses in agricultural systems is abiotic stress^5^, which limits plant production^6^. Among abiotic stresses, nutritional disorders stands out, because the capacity of plants to uptake nutrients depends on their availability in the soil, and the lack of nutrients can affect essential components of plant metabolism^7^.

In the above described nutritional stress disorder scenario, the application of beneficial elements such as silicon (Si) has been indicated as promising. Although it is not considered a nutrient, Si is responsible for plant protection under abiotic stress conditions^8–10^. Still, to date, most studies have concentrated more on the attenuation of ammonium, nitrate, and metal excess toxicity (e.g., in cucumber^11^) than on their deficiency in plants, whereas studies indicating the benefit of Si in plant nutrient deficiency have mostly focused on nutrient deficiency in major crops, such as nitrogen deficiency in rice^12^, potassium deficiency in sorghum^13^, magnesium deficiency in corn^14^, and sulfur deficiency in barley^15^.

Regarding the current knowledge about plant nutrient requirements, it is known that nutrients perform essential and specific functions in plant metabolism. Thus, when a nutrient is not present in adequate amounts in the soil, it becomes limiting, which in turn promotes its deficiency in the cells and certain metabolic changes in plants. Despite recent studies involving mineral nutrition for herbaceous performance of the quinoa^16^, research on mineral nutrition is still insufficient for the crop.

The symptoms of deficiency or toxicity are usually specific to each nutrient and are influenced by the severity, species, and variety, besides environmental factors^17^. However, researches addressing nutrient deficiency in quinoa crop, and how Si application influences nutrient deficiency, are still scarce.

The beneficial effect of silicon on plants under stress conditions caused by nutrient deficiency has been attributed to the protection of the plant photosynthetic system^18^, increased production of chlorophylls^19^, stimulation of antioxidant systems, and improved physical integrity of membranes^18^, in addition to maximized nutrient uptake^8,13,20,21^.

In the present study, the objective was to evaluate the symptoms of nitrogen, phosphorus, potassium, calcium, and magnesium deficiencies under Si fertilization in terms of tissue, physiological, and nutritional effects of quinoa plants. The hypotheses of the study are that: (a) in quinoa plants, the deficiency of macronutrients would result in physiological disorders and symptoms at the tissue level according to the function of each nutrient in the plant metabolism; (b) the application of Si could mitigate the stress caused by nutrient deficiency in quinoa plants by increasing the uptake of missing nutrients as well as by increasing the synthesis of pigments and protecting the photosynthetic apparatus.

If these hypotheses were confirmed, it would allow a better understanding of the benefits of Si for plant nutrient deficiency, and it would consequently increase the sustainability of quinoa crop production, especially in areas characterized by low soil fertility. In this way, our objective was to evaluate the symptoms in terms of tissue, physiological and nutritional effects of quinoa plants submitted to stress by nitrogen (N), phosphorus (P), potassium (K), calcium (Ca), and magnesium (Mg) deficiencies under Si fertilization.

## Material and methods

### Experimental conditions

**Figure 1:**
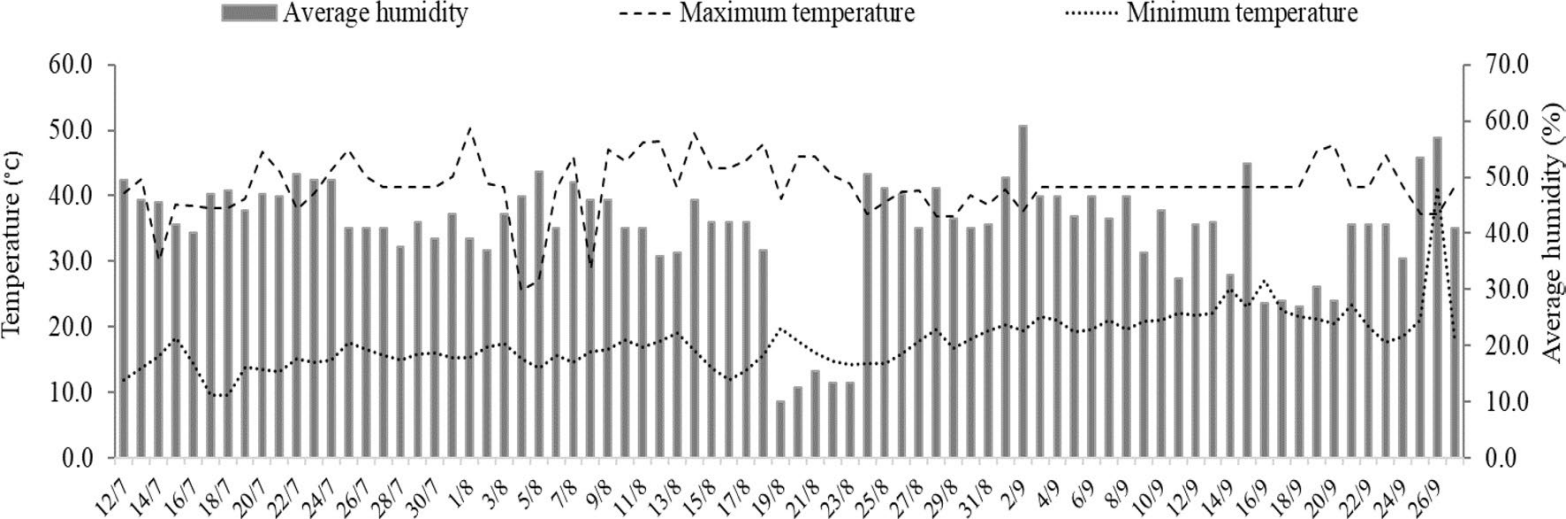
Minimum and maximum temperature (°C), and relative air humidity (%) recorded using a thermo-hygrometer during the experimental period inside the greenhouse. Software used to create this Figure was Microsoft Excel (v1804, https://www.microsoft.com).

The experiment was carried out in a greenhouse between July and September 2019. The seeds used belong to the BRS Piabiru cultivar, marketed by Embrapa (Brazilian Agricultural Research Corporation). During the experimental period, the air humidity and maximum and minimum temperature were recorded using a thermo-hygrometer (Fig. 1). There was a wide variation in the relative air humidity (40.9 ± 18.9%), maximum temperature (41.4 ± 12.3 °C), and minimum temperature (31.5 ± 9.6 °C), and the temperatures reached values that were above the optimal values for quinoa growth (− 4 °C and 38 °C)^22^. All plant studies were carried out in accordance with relevant institutional, national or international guidelines and regulation.

Seeding was performed in two trays with 128 cells (volume of 26.6 mL each cell), containing washed sand as a substrate. After sowing, the seeds were irrigated with distilled water until the emergency period. Then, six seedlings were transplanted into 1.7 dm^3^ polypropylene pots filled with 1.5 dm^3^ of washed sand. After transplantation, the plants were irrigated with a complete nutritional solution^23^ at 10% of the concentration, as indicated by Hoagland and Arnon^23^, with a change in the iron source from Fe-EDTA to Fe-EDDHMA. The adjustment of the pH value to 5.5 ± 0.1 was performed by adding NaOH (1 mol L^−1^) or HCl (1 mol L^−1^) solution.

### Experimental design

The experiment was carried out in a 6 × 2 factorial scheme, and it included the following treatments: control (complete nutritional solution); omission of N (4 mmol L^−1^ throughout the experimental period); P (6 mmol L^−1^, from 10 days after transplanting, DAT); K, Ca, and Mg combined with Si (1.5 mmol L^−1^) and without Si. Treatments were arranged in randomized blocks with nine replicates. The nutrient solution for the treatments was prepared according to Hoagland and Arnon^23^.

### Starting treatments and Si supply

**Figure 2:**
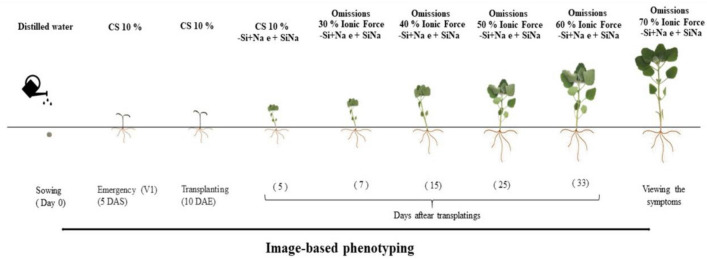
Schematic representation of the experimental project. After the seeds germinated, the seedlings were grown in different ionic forces of Hoagland solution (Hoagland and Arnon^23^). *CS* complete solution, *DAS* days after sowing, *DAE* days after emergence. Software used to create this Figure was Microsoft Word (v1804, https://www.microsoft.com).

At 5 DAT, Si supply was started using a complete nutritional solution (Fig. 2). The source of Si was sodium silicate (188.4 g L^−1^ of Si; 121 g L^−1^ of Na_2_O, and pH 12). Si was added to the nutrient solution, and the pH value was adjusted to 5.5 ± 0.2 with HCl (1.0 mol L^−1^) or NaOH (1.0 mol L^−1^) solutions, and it was immediately supplied to the plants. The additional application of 49.8 mg L^−1^ of NaCl was performed in the treatments without Si to balance the Na among treatments.

As the source of Si used was sodium silicate, it was necessary to balance the sodium between treatments, due to the importance of this element for quinoa crop^24^. For this purpose, 19.75 mg L^−1^ of Na, in the form of NaCl was supplied via a nutrient solution during the entire experimental period, starting together with the Si supply.

At 7 DAT, it was started nutrient omission in the nutrient solution and increased its concentration to 30% of that indicated by Hoagland and Arnon^23^ for 7 days, then to 40% for 10 days, 50% for 8 days, 60% for 13 days, and 70% by the end of the experimental period. Once a week, the substrate was washed to avoid salinization. Deionized water was left to drain from the substrate, and the nutrient solution was applied again after 2 h.

### Phytosanitary treatments

For pest control, insecticide application (Thiamethoxam 141 g L^−1^ + Lambdacyhalothrin 106 g L^−1^) at a rate of 1.5 mL L^−1^ was performed at 38 DAT. Furthermore, acaricide applications (Fenpiroximato, 50 g L^−1^ and Abamectina, 18 g L^−1^) were performed twice a week from 40 DAT until the end of the experiment, at a rate of 1 mL L^−1^.

### Green color index, photosystem II quantum efficiency, and pigment content

At 40 days of growing and 10 days from treatment application, the symptoms of N, P, K, and Mg limitation were characterized at the tissue level. At 60 days of growing and 35 days from the treatment application, the symptoms of Ca limitation were characterized. As soon as the symptoms were characterized, physiological evaluations of the green color index (GCI), fluorescence and quantum efficiency of photosystem II (Fv/Fm), and chlorophyll and carotenoid contents were performed. The GCI and pigment content were measured in the fourth leaflet from the apex of the main stem. GCI was measured using a chlorophyllometer (Opti-Sciences, CCM-200), with five readings per plant.

For pigment analysis, six leaf discs (6 mm) obtained from the middle third of the leaf limb were collected. The analyses were performed according to the methodology proposed by Lichtenthaler^25^ using a Beckman DU 640 spectrophotometer at the following wavelengths: 663 nm for chlorophyll a, 647 nm for chlorophyll b, and 470 nm for carotenoids. Their contents were defined in relation to the fresh mass of each disc.

The Fv/Fm was obtained from the chlorophyll fluorescence measurement using a fluorometer (Opti-Sciences, Os30P). The measurements were carried out between 7:30 a.m. and 9:30 a.m. using one plant per pot, and we measured the middle third of the plant, evaluating the fourth leaflet from the apex of the main stem. For this measurement, the sampled region was placed in the dark for adaptation at least 30 min before the excitation of the red-light pulse of 1 s. The evaluated parameters were F0 (minimum fluorescence for chlorophyll excitation) and Fm (maximum fluorescence for chlorophyll excitation). From these parameters, F0/Fm (basal quantum yield) and Fv/Fm (quantum efficiency of photosystem II) were obtained. The leaf area was also evaluated using the equipment Li-Cor model L1-3000 and was measured in cm^2^.

Later, the leaves, stems, and roots were washed with water, detergent solution (Extran 0.1%), acid solution (HCl 0.1%), and deionized water, and then packed in paper bags and placed in dry conditions in a forced air circulation oven at 65 ± 5 °C until a constant mass was obtained. After drying, the samples were weighed on a precision scale (0.001) to obtain the shoot dry mass (leaves + stem) and root dry mass.

### Electrolyte leakage index

For determining the cellular leakage index, six leaf discs (6 mm) were removed from the middle third of the plant, which had new and fully developed leaves. The discs were packed in a beaker with 20 mL of deionized water at ambient temperature for 2 h. After this period, the initial electrical conductivity reading (EC1) was performed using a manual conductivity meter (TDS-3 digital measurer). Afterward, samples were taken to autoclave for 20 min at a temperature of 121 °C, and after cooling, a new conductivity reading was performed to determine the final conductivity (EC2). To estimate the leakage, the following formula^26^ was used: EC1/EC2 × 100.

### Nutritional assessments and Si content

After weighing the material, the samples were ground in a Wiley type mill in order to determine the Si, N, P, K, Ca, Mg contents in plant shoots. For quantifying Si, the plant material was digested by the wet digestion method, using hydrogen peroxide (H_2_O_2_) and sodium hydroxide (NaOH)^27^, and the determination of the Si content was performed after the reaction of samples with ammonium molybdate, followed by a spectrophotometer reading at 410 nm^28^. For quantifying the macronutrient contents (N, P, K, Ca, Mg), the Bataglia methodology^29^ was adopted. The accumulation of Si, N, P, K, Ca and Mg was calculated by multiplying dry mass and element contents, and it was expressed as mg per shoot.

### Statistical analysis

Data were submitted to analysis of variance by the F test, and when significant, the mean comparison was performed by the Tukey test at 5% probability using the Sisvar software^30^. Later, a heatmap was built for a view of the interrelationship between the treatments and the variables evaluated. For that, the variables were standardized and the Euclidean distance between the treatments was used. The “pheatmap” library of the R software was used.

## Results and discussion

Although all treatments were subjected to high air temperature (Fig. 1), it did not influence the development of the quinoa plants. It is worth noting that the high temperature occurred only in 2 h during the day (between 12 and 14 pm) and at some punctual moments during the entire growth cycle. However, it is known that the crop is tolerant to high temperatures^31^, and recent research shows that quinoa can complete its cycle even under conditions of high air temperatures (> 40 °C)^32^^.^

### Nitrogen (N)

**Figure 3:**
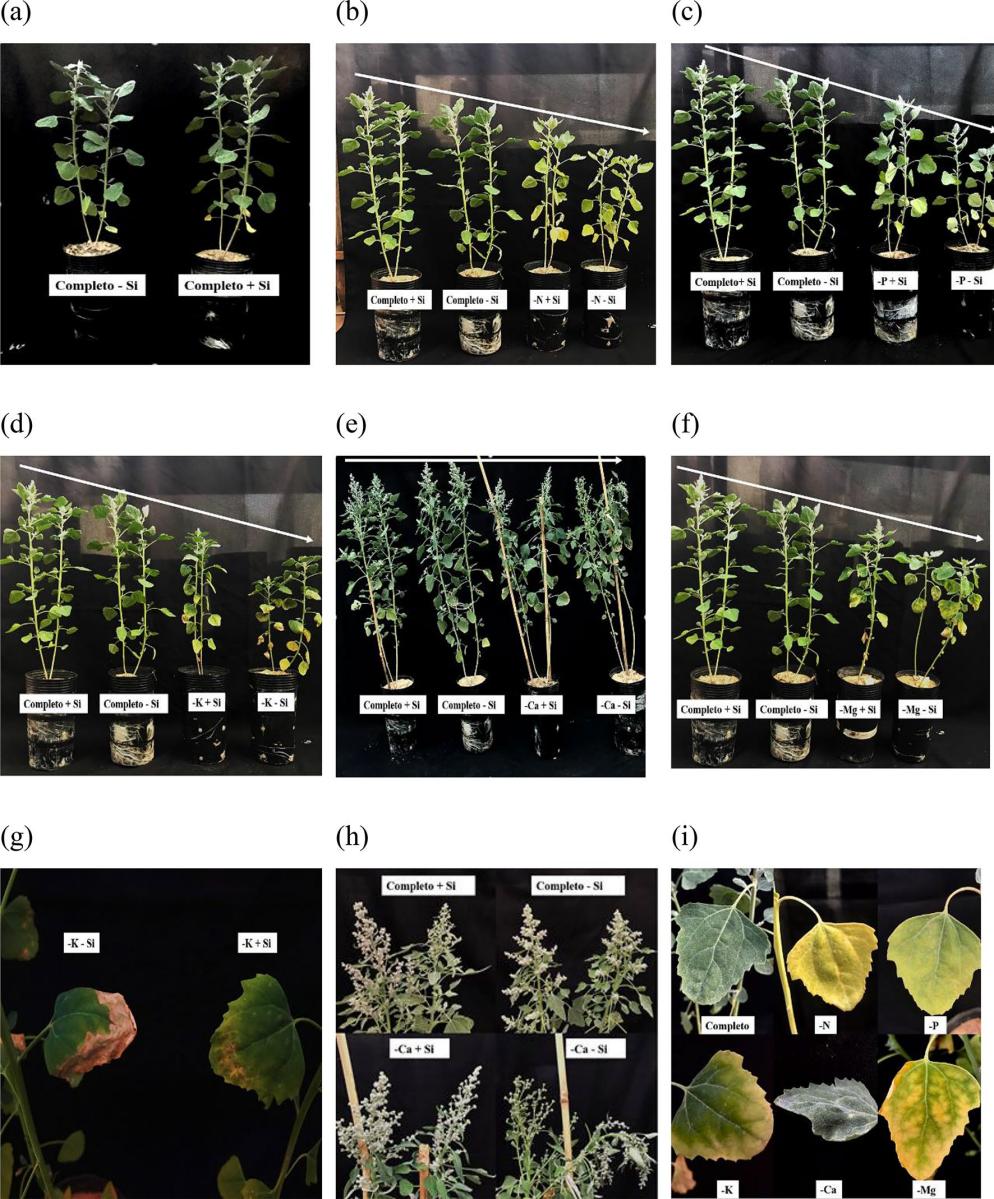
Symptomatology of quinoa plants submitted to Hoagland solution (Hoagland and Arnon^23^) under omission of macronutrients in the absence and presence of Si, (**a**) complete solution, (**b**) nitrogen omission, (**c**) phosphorus omission, (**d**) potassium omission, (**e**) calcium omission, (**f**) magnesium omission, (**g**) quinoa leaves under potassium omission, (**h**) quinoa inflorescence under calcium omission, (**i**) comparison of macronutrient symptoms on quinoa leaves. Software used to create this Figure was Microsoft Word (v1804, https://www.microsoft.com).

As a consequence of N omission to quinoa plants, the symptom observed was chlorosis in older leaves (Fig. 3i). N omission resulted in a decrease of 92% in N accumulation under absence of Si and 88% under presence of Si compared to the N accumulation of plants grown in the complete solution; thus, the symptoms of deficiency appeared. As the symptoms evolved, chlorosis became apparent in the upper portion of the plants, but was not markedly high due to the high mobility of nutrients through the phloem^33^ (Fig. 3b).

The process of depigmentation of N-deficient leaves can be considered as consequence of nutrient redistribution within plant metabolism. Due to N deficiency, the proteolysis of Rubisco enzyme and other chloroplast proteins allows the release and relocation of the nutrient to the new organs^34^ with the aim to ensure plant growth and development.

**Figure 4:**
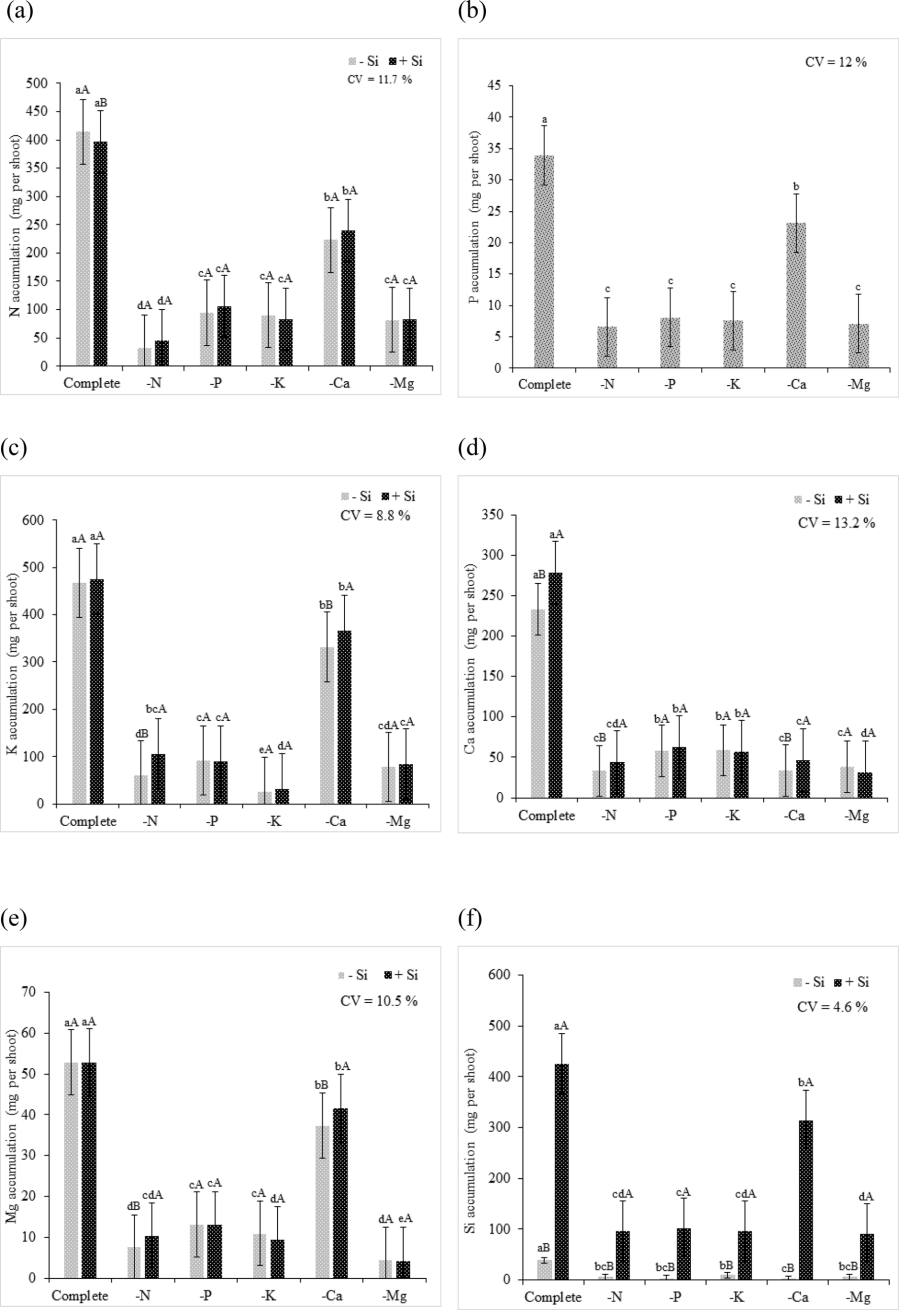
Nutrient accumulation in quinoa plants in a Hoagland solution (Hoagland and Arnon^23^) under omission of macronutrients in the absence (− Si) and presence of silicon (+ Si), (**a**) N accumulation (mg per plant), (**b**) P accumulation (mg per plant), (**c**) K accumulation (mg per plant), (**d**) Ca accumulation (mg per plant), (**e**) Mg accumulation (mg per plant), and (**f**) Si accumulation. Equal lowercase letters show a similarity between omission and complete treatments by Tukey’s test at 5% probability. Equal uppercase letters show a similarity between treatments (− Si and + Si) by Tukey’s test at 5% probability. Software used to create this Figure was Microsoft Excel (v1804, https://www.microsoft.com).

Regarding the accumulation of nutrients in quinoa plants, the Si supply resulted in an increase in Si contents in plant shoots (Fig. 4f), and it also promoted a higher accumulation of K, Ca, and Mg compared to that in the plants that did not receive Si (Fig. 4c–e). The increased accumulation of K under Si supply was possibly a consequence of the activation of H^+^ ATPase in the membranes, when Si was added to the nutrient solution^35^, as Si improves the uptake and translocation of K^+^^36–39^. On the other hand, Ca accumulation was possibly a consequence of the increased Ca inflow and outflow into the cytosol through Ca carrier channels located in the membrane^40^. In a study assessing drought stress in corn, Kaya et al.^40^ reported an increase in Ca accumulation in unstressed plants after Si addition. Likewise, our findings suggested that the Si addition resulted in higher Ca accumulation when a complete nutrient solution was applied to quinoa plants.

**Figure 5:**
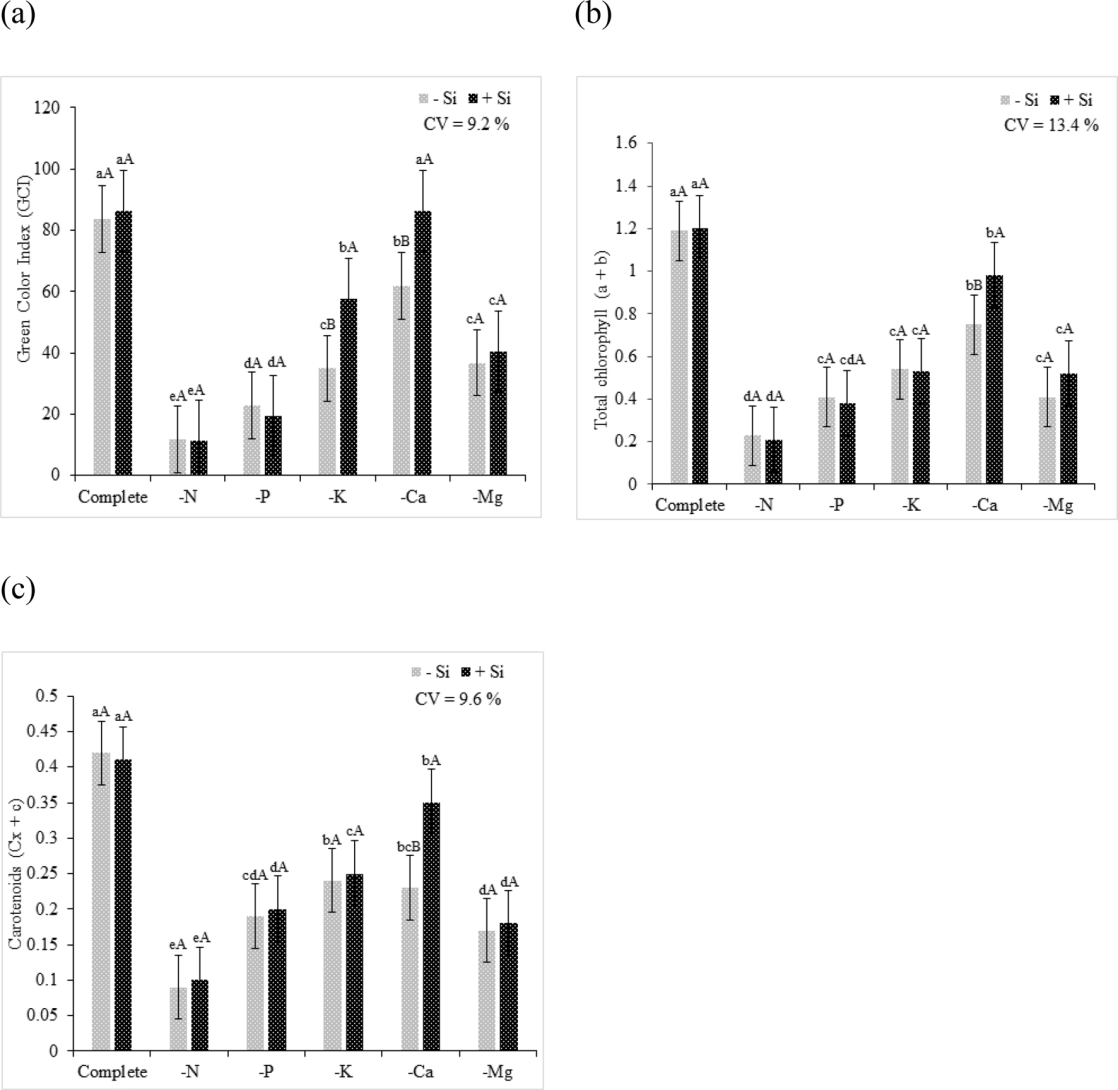
(**a**) Green color index (GCI), (**b**) chlorophyll a + b, and (**c**) carotenoids of quinoa plants in a Hoagland solution (Hoagland and Arnon^23^) under omission of macronutrients in the absence (− Si) and presence of silicon (+ Si). Equal lowercase letters demonstrate a similarity between the omitted and complete treatments by Tukey’s test at 5% probability. Equal uppercase letters show a similarity between treatments (− Si and + Si) by Tukey’s test at 5% probability. Software used to create this Figure was Microsoft Excel (v1804, https://www.microsoft.com).

N deficiency resulted in a decrease in the GCI of quinoa plants (Fig. 5a) due to the decrease in pigment content. The decrease in chlorophyll content a + b and cx + c was observed in quinoa plants that experienced N deficiency regardless of the Si supply (Fig. 5b,c). The omission of the macronutrient caused a decrease of 81% and 83% in chlorophyll a + b content and 79% and 76% of cx + c, under absence and presence of Si, respectively, of plants grown in complete solution. This effect was related to the structural role of N in the chlorophyll molecule^41^. In some crop species, leaf chlorophyll content is positively correlated with N concentration, and 70% of the N contained in leaves participates in the synthesis and chemical structuring of chlorophylls^42^.

**Figure 6:**
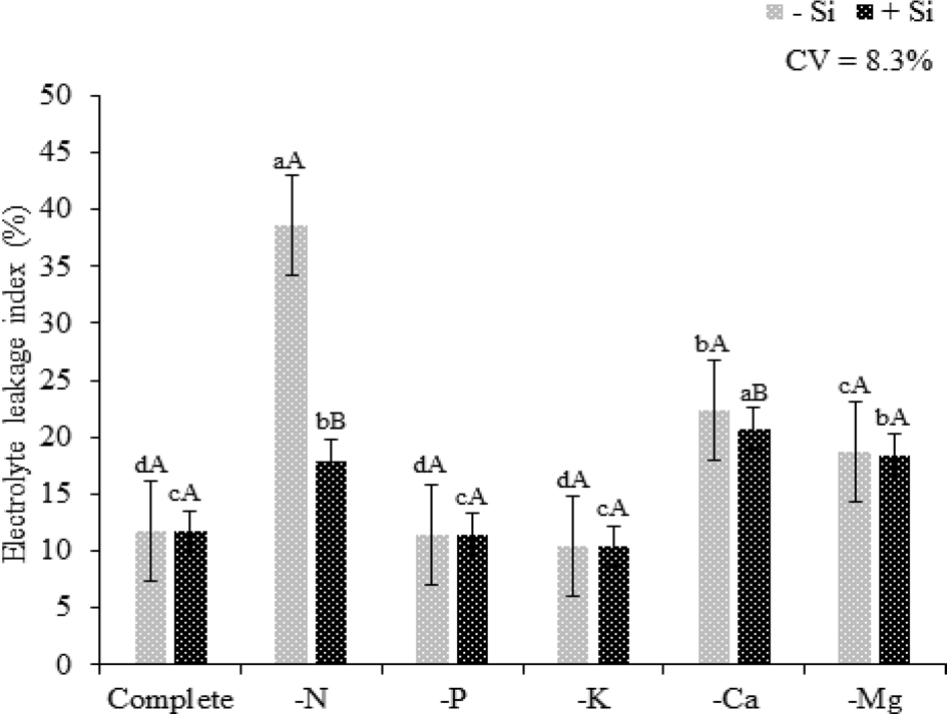
Electrolyte leakage index of quinoa plants in a Hoagland solution (Hoagland and Arnon^23^) under omission of macronutrients in the absence (− Si) and presence of silicon (+ Si). Equal lowercase letters demonstrate a similarity between the omitted and complete treatments by Tukey’s test at 5% probability. Equal uppercase letters show a similarity between treatments (− Si and + Si) by Tukey’s test at 5% probability. Software used to create this Figure was Microsoft Excel (v1804, https://www.microsoft.com).

In the present study, regardless of the Si supply, N deficiency resulted in a higher electrolyte leakage index of quinoa plants compared to that of quinoa plants grown in the complete solution. However, the Si supply in the solution mitigated the deleterious effects of nutrient deficiency by reducing electrolyte leakage (Fig. 6). Previous reports have indicated that Si forms complexes with cell structural polymers, such as pectins and callose^43^, and cross-links with lignins and carbohydrates by associations with phenolic acids or aromatic rings^44^, thus contributing to cell membrane structuring.

**Figure 7:**
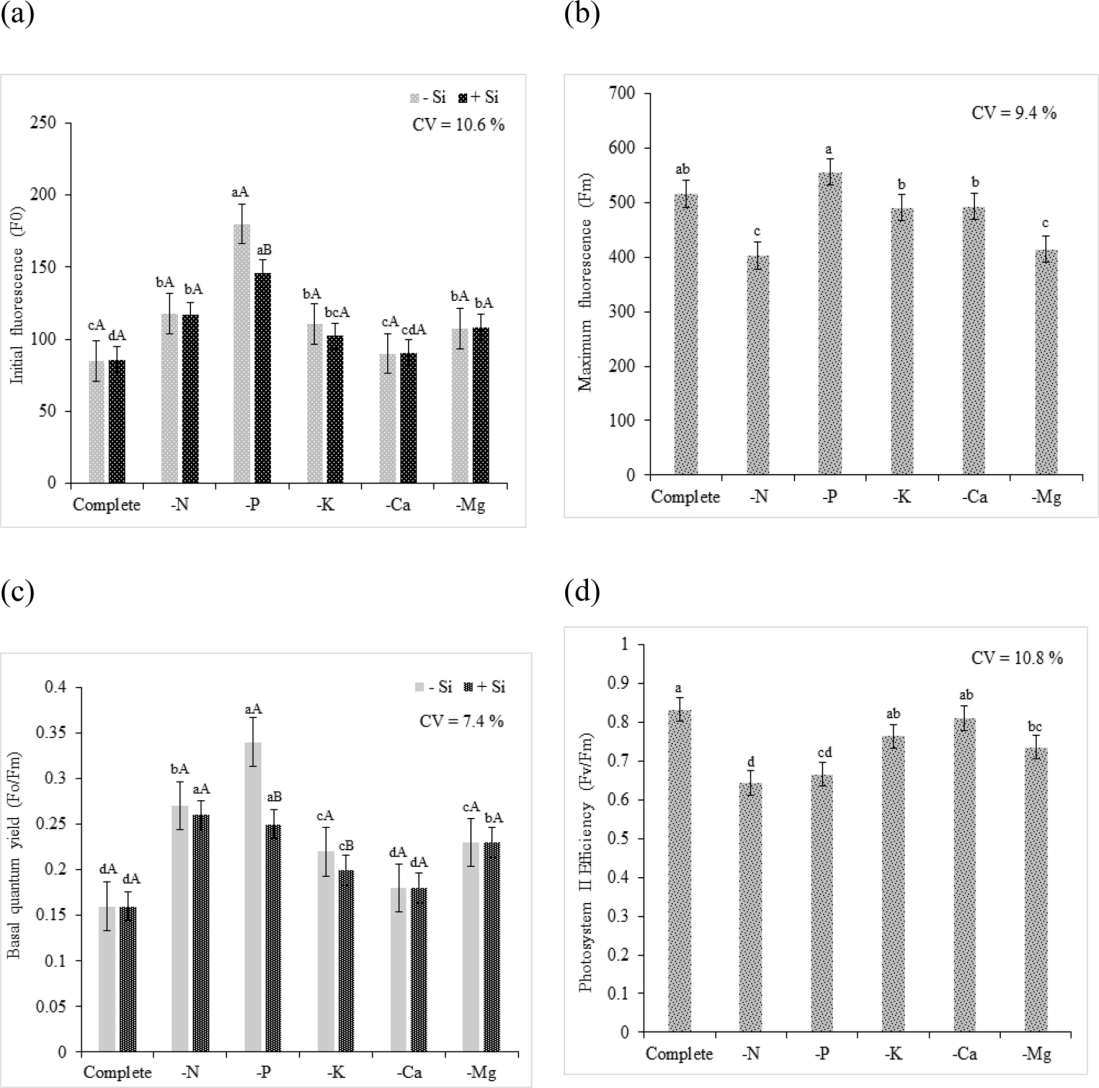
Physiological evaluations of quinoa plants in a Hoagland solution (Hoagland and Arnon^23^) under omission of macronutrients in the absence (− Si) and presence of silicon (+ Si, (**a**) Initial fluorescence (Fo), (**b**) Maximum fluorescence (Fm), (**c**) basal quantum yield of Photosystem II, and (**d**) Photosystem II Efficiency. Equal lowercase letters demonstrate a similarity between the omitted and complete treatments by Tukey’s test at 5% probability. Equal uppercase letters show a similarity between treatments (− Si and + Si) by Tukey’s test at 5% probability. Software used to create this Figure was Microsoft Excel (v1804, https://www.microsoft.com).

In the present study, there was an increase in F0 in plants in the N omission treatment, regardless of the Si supply, compared to that of plants grown in the complete solution (Fig. 7a). Baker and Rosenqvist^45^ reported that increased F0 indicates the destruction of the photosystem II (PSII) reaction center, chlorophyll (P680), or a decreased capacity of energy transfer from the antenna complex to the PSII. These authors also stated that this occurs when quinone (QA, which is a primary electron receptor) is oxidized and when the reaction center (i.e., chlorophyll) is in an “open” state, indicating urgency of the activation of photochemical reactions.

The Si supply in the solution did not have a significant influence on Fm (Fig. 7b). The N deficiency promoted a decrease in the Fm rate compared to that of plants grown in the complete solution. Baker and Rosenqvist^45^ stated that the increased maximum fluorescence intensity (Fm) denotes the state in which PSII reaction centers are unable to increase photochemical reactions. This condition indicates that fluorescence has reached its maximum capacity by the completely reduced condition of quinone (QA), and in which the reaction center (P680) is in a “closed” state, thereby affecting the transport of electrons to photosystem I (PSI).

Regarding F0/Fm under N omission, it was observed higher rates of yield compared to that of plants cultivated in the complete solution. The latter had a production of 0.16, regardless of Si supply, whereas the former had a yield of 0.27 under absence of Si and 0.26 under presence of Si, indicating stress conditions (Fig. 7c). According to Rohácek^46^, an increased F0/Fm ratio is indicative of stress, and normal F0/Fm ratio values close to 0.14 and 0.20 are commonly described in literature.

N deficiency affected the Fv/Fm in quinoa plants, as PSII efficiency was 0.644, which was 23% lower than that of the plants grown in the complete solution (whose Fv/Fm ratio was 0.833; Fig. 7d). One study reported that the Fv/Fm ratio of plants under normal conditions was between 0.85 and 0.75, and that values lower than 0.75 would indicate inhibitory PSII conditions^47^. Thus, our finding showed that N deficiency affects quinoa photosynthetic efficiency because N is an essential component for the functioning of proteins and chlorophyll, and, consequently, photosynthesis^48^. Photosynthetic capacity is associated with the N content in plant metabolism, mainly because the tilacoids and proteins in the Calvin cycle account for the highest proportion of N in plant metabolic components^49^.

**Figure 8:**
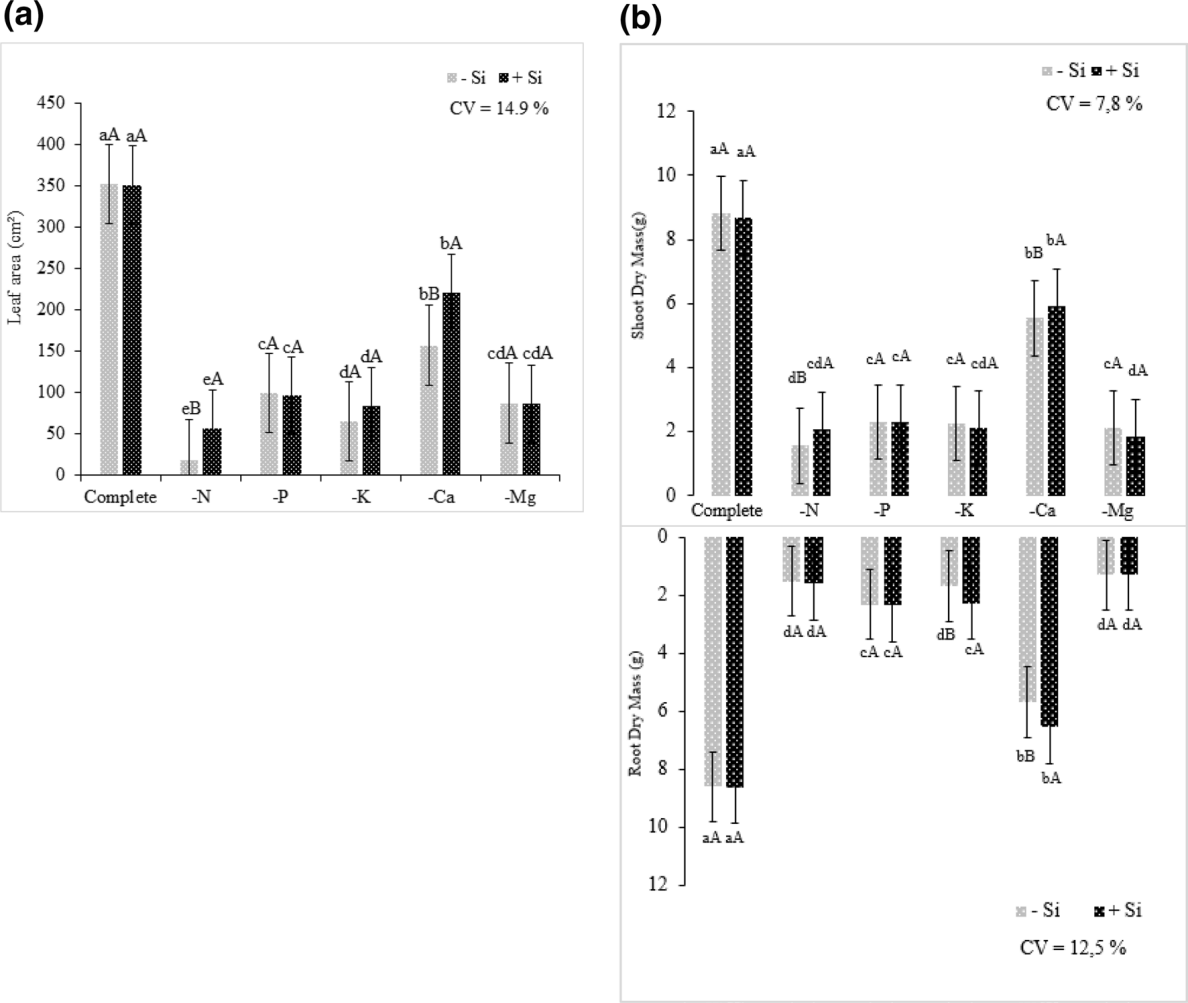
Biomass production of quinoa plants submitted to Hoagland solution (Hoagland and Arnon^23^) under omission of macronutrients in the absence (− Si) and presence of silicon (+ Si), (**a**) leaf area, (**b**) shoot and root dry mass. Equal lowercase letters demonstrate a similarity between the omitted and complete treatments by Tukey’s test at 5% probability. Equal uppercase letters show a similarity between treatments (− Si and + Si) by Tukey’s test at 5% probability. Software used to create this Figure was Microsoft Excel (v1804, https://www.microsoft.com).

Regarding the development of N-deficient plants, the lower pigment content and consequently lower photosynthetic rate resulted in a smaller leaf area than that of plants grown in the complete solution (Fig. 8a). However, the Si supply had a positive effect on plant development, resulting in a leaf area 68% higher compared to that of plants grown under the absence of Si.

Regarding plant development, for the shoot dry mass, it was evident that the N omission resulted in the decrease in the biomass of plants grown with or without Si (Fig. 8b). However, the presence of Si in the nutrient solution had beneficial effects on plant development. In the N omission treatment, higher shoot biomass was observed in plants that were supplied with Si than in those that were not supplied with Si. This effect was possibly because Si mitigated N nutritional stress.

It was also evident that the N omission resulted in a decrease in root dry mass. However, the presence of Si was not sufficient to influence root growth and mitigate severe deficiency effects. N was one of the macronutrients that was most limiting to the growth of quinoa plants, with a decrease of 82% in both shoot and root dry mass under absence of Si, and a decrease of 76% and 81% in the shoot and root dry mass under Si presence, respectively, compared to those values in plants without N omission. These findings suggested that the amount of N required by a given plant, including quinoa, is a very important factor in plant growth, as this nutrient is of great relevance for plant growth^50^.

However, the Si supply improved plant performance under stress conditions, resulting in a larger leaf area and dry matter biomass than that of plants under stress conditions and not supplied with Si. Thus, it became evident that the addition of the beneficial element, in this case Si, resulted in better performance of quinoa plants submitted to N deficiency stress.

### Phosphorus (P)

Regardless of the presence of Si, quinoa plants showed visual symptoms of deficiency, such as pale green leaves and darker green upper leaves. As the symptoms progressed, the lower leaves became necrotic and the upper leaves became pale green. The slower development of nutrient-deficient quinoa plants than that of plants grown in the complete solution was also evident (Fig. 3c,i). These symptoms were the results of lower P availability; in these plants, a 76% lower accumulation nutrient content compared to that of plants grown in the complete solution was observed (Fig. 4b). However, the supply of Si resulted in higher shoot Si contents than that of plants not supplied with Si (Fig. 4f).

The progress of symptoms evidenced the high P mobility in the phloem. In nutrient deficiency conditions, P redistribution occurs in order to meet the demand of developing tissues^50^. Regarding the GCI, the P-deficient plants had low intensity of coloration compared to that of plants in the complete solution, regardless of the presence of Si (Fig. 5a).

The decrease in chlorophyll a + b and cx + c in plants as a consequence of P omission confirmed that the decreased pigment rate resulted in lower green color intensity (Fig. 5b,c). Pigment degradation can explain the depigmentation of leaves under P omission conditions, and as a result, there was a decrease in photosynthetic activity caused by lower ATP synthesis and Rubisco regeneration^51^.

Regardless of the Si supply, P omission did not result in an increase in electrolyte leakage index; the plants under P omission had similar electrolyte leakage index as that of the plants grown in the complete solution (Fig. 6). By analyzing F0 regardless of the presence of Si, the P deficiency increased photosystem energy loss compared to this loss in the plants grown in the complete solution. However, the presence of Si in P-deficient plants decreased the energy loss by fluorescence by compared to that of plants that did not receive Si, revealing a supposed beneficial effect of Si on the photosynthetic system of quinoa plants (Fig. 7a). The P omission resulted in the highest Fm, but it was not statistically different from that of plants grown in the complete solution (Fig. 7b).

The F0/Fm in the P-deficient plants resulted in higher quantum basal yield than that in plants grown in the complete solution. The use of Si resulted in a significant difference, as an average quantum basal yield of 0.35 was recorded under Si absence, whereas an average quantum basal yield of 0.25 was recorded under Si supply. However, although the mean was not standardized as normal, Si showed beneficial effects on this variable, and it was able to mitigate possible deleterious effects of P deficiency (Fig. 7c).

In plants grown with P omission, the Fv/Fm ratio was 0.666, which was 20% lower than that in quinoa plants grown in the complete solution, which had the Fv/Fm ratio of 0.833 (Fig. 7d). The Fv/Fm ratio can indicate perturbations in the photosynthetic system as a consequence of environmental and biotic stresses^52^. When this ratio decreases, it indicates inhibition of photochemical activity or deviation in absorbed luminous energy, evidencing damage in the photosynthetic apparatus, and consequently the decrease in quantum efficiency^53^.

Regardless of the presence of Si, the leaf area of plants under P omission showed a decrease compared to that of plants grown in the complete solution, demonstrating the importance of this macronutrient for plant development (Fig. 8a). The slower development of the plants grown under P absence occurred due to the metabolic function of P in the process of energy transfer and storage^54^. P deficiency affects the energy bonds in plant metabolism, as this nutrient is a component of NADP and ATP^55^.

In summary, the findings on the influence of P deficiency on shoot and root biomass, regardless of the Si supply, emphasized the negative effects of P deficiency on plant metabolism, evidencing that the nutritional stress limited the development of P-deficient plants compared to those grown in the complete solution (Fig. 8b). The scarcity of energy in plant metabolism compromises the biosynthesis processes^41,56^. Consequently, there is a decrease in the formation of phospholipids, which are essential cell membrane components. As a result, the synthesis of new cells decreases^57^, resulting in limited plant development.

### Potassium (K)

Quinoa plants had a decrease in K accumulation of 94% and 93% under the absence and presence of Si, respectively, compared to that of plants cultivated in the complete solution (Fig. 4c). The Si supply resulted in higher Si contents in plant shoots than that in the shoots of plants not supplied with Si (Fig. 4f).

The symptoms of K deficiency included chlorosis only at the margins of older leaves, which was followed by necrosis (Fig. 3d,g,i). Low K availability promotes the decrease in protein synthesis, which is reflected in the accumulation of decarboxylated amino acids. This increases putrescine content, whose high concentrations accentuate cellular imbalance and promote marginal necrosis of plant tissues^58^.

The GCI of K-deficient plants, regardless of the presence of Si, had lower intensity than that of plants grown in the complete solution (Fig. 5a). However, the Si supply reduced the depigmentation damage caused by K deficiency. This may have occurred due to the beneficial effect of Si in protecting the photosynthetic apparatus, which was corroborated by the decrease in F0/Fm the efficiency of Fv/Fm, allowing an efficient photosynthetic metabolism even with the nutrient deficiency (Fig. 7c,d).

Regardless of the presence of Si, K omission decreased the content of chlorophyll a + b and cx + c compared to that in plants cultivated in the complete solution. Despite the Si supply, the beneficial element was unable to favor the increased rate of these photosynthesizing pigments (Fig. 5b,c).

The electrolyte leakage in plants under K deficiency showed no significant difference from that of plants under the complete treatment, regardless of Si supply (Fig. 6). This was because unlike N and P, K does not have a structural role in plant metabolism. Its primary function in the plant metabolism is enzymatic activation, and it is required for the function of more than 60 enzymes^41^.

In K-deficient plants, regardless of the presence of Si, the F0 was higher than that in plants grown in the complete solution. Despite this, the Si supply was unable to increase the performance of K-deficient plants (Fig. 7a). The increase in F0 in K-deficient plants could be attributed to the damage to the PSII light receptor complex or the decrease in the transfer of excitation energy from the light collector system to the reaction center^45,59,60^, which were consequences of stress conditions. Fm did not differ statistically between K-deficient plants and plants grown in the complete solution (Fig. 7b).

The F0/Fm of K-deficient plants was significantly higher than that of plants grown in complete solution, and the former had increased quantum basal yield. The use of Si in the K omission solution had a significant positive effect; the absence of Si provided a quantum yield of 0.22, evidencing the stress condition, whereas in the presence of Si, the observed quantum yield was of 0.20, corresponding to the range indicated as normal (Fig. 7). These results demonstrated a possible beneficial effect of Si on preserving the photosynthetic apparatus in abiotic stress conditions. As the Fv/Fm ratio of K-deficient plants was 0.764, there was no statistical difference between these plants and the plants grown in the complete solution (Fig. 7d), revealing a photosynthetic efficiency of K-deficient plants. The positive effect of of Si addition on the K-deficient plants was also verified by Chen et al.^13^ in sorghum, where under nutritional stress, plants treated with Si presented higher photosystem II efficiency than that of untreated plants. The authors reported that the Si supply through the root system resulted in improvements in plant development and photosynthetic activity rates.

In the present study, the leaf area was reduced as a consequence of K deficiency regardless of the Si supply (Fig. 8a). Regardless of the presence of Si, the biomass of quinoa plants was affected by K deficiency, with a decrease of 74% and 76% in the absence and presence of Si, respectively, compared to that of plants grown in the complete solution (Fig. 8b). However, the presence of Si in K-deficient plants had positive effects on root development compared to that of plants in the absence of Si.

The lack of K results in lower rates of carbohydrate translocation from shoots to roots, thus reducing root development^41^. Therefore, the findings showed that K deficiency reduced the shoot biomass, and the Si supply of K-deficient plants resulted in increased root biomass.

### Calcium (Ca)

The quinoa plants cultivated in the Ca omission presented a decreased Ca accumulation of 86% and 83%, in the absence and presence of silicon, respectively, compared to quinoa plants cultivated in complete solution (Fig. 4d). The Si supply provided greater Si accumulations in the shoot (Fig. 4f).

The symptoms of Ca deficiency are characterized by poor formation of the growth points, the youngest leaves, sprouting and inflorescence had irregularities (Fig. 3e,h,i). Studies carried out by Silva et al.^61^ in the jatropha crop, and Miranda et al.^62^ in cowpea (*Vigna unguiculata* (L.) Walp) also found that calcium deficiency leads plants to grow with disorganized leaves and wavy edges. This occurs because Ca presents structural function, conferring pectin stability on the cell wall of the leaves^63,64^.

Despite the decrease of Ca uptake by cultivated plants in the absence of the nutrient, the Si supply provided higher uptake efficiency of the missing element. There are several mechanisms that explain the benefits of using Si in the relief of the nutrient deficiency of plants, increasing the remobilization or uptake of the deficient nutrient^13,65–68^. This reveals the beneficial effect of Si in improving the efficiency of the stressed plant for missing element uptake, resulting in better performance in using the nutrient needed. The presence of Si under Ca restriction provided higher efficiency in K and Mg uptake, which was evidenced by the higher accumulation of these macronutrients. Moreover, the presence of Si provided greater efficiency in K and Mg uptake, which was evidenced by the higher accumulation of these nutrients under restriction.

The GCI under absence of the Si is indicative of damages caused by the Ca deficiency, since there was a decrease in the green coloration under these conditions. On the other hand, in Ca deficient plants under Si supply, GCI was similar with the plants grown in complete solution (Fig. 5a). Using Si contributed to the mitigation of Ca deficiency by increasing the chlorophyll content a + b and cx + c (Fig. 5b,c). The findings reaffirm the benefits of Si on stressed plants by increasing the chlorophyll^69^ content or by protecting the degradation of chlorophyll^70^ in stress situations.

Ca deficiency regardless of the presence of Si showed higher electrolyte leakage index compared to quinoa plants grown in complete solution (Fig. 6). This occurs because Ca is a nutrient with remarkable importance for cellular structuring, since cation binds to the pectins, forming the calcium pectates, which are compounds that give stability to the plasmatic membrane^71^, besides the presence of Ca reduce the action of the enzymes Polymethylesterase (PME) and Polygacaracturonase (PG)^67^, which are enzymes that oxidize pectin. However, the Si supply in the nutritive solution under Ca omission provided decrease in electrolyte leakage index, which can be related to better activity of antioxidant enzymes during the oxidative stress^72–74^.

For Ca deficient quinoa plants, regardless of the presence of Si, no changes were found for photosynthetic variables. The variables F0, Fm, F0/Fm and Fv/Fm did not present significant differences regarding the quinoa plants grown in complete solution (Fig. 7a–d). This finding evidence that Ca deficient plants can present an efficient transport of electrons by PSII in non-oxidized state^75^.

The leaf area showed a decrease in Ca omission plants compared to plants grown in complete solution. In plants grown in Ca deficiency, the addition of Si promoted an increase of 140.49% in the leaf area index in comparison to quinoa plants submitted to Ca omission in the absence of the element (Fig. 8a). The Si supply may have contributed to mitigating the Ca deficiency stress by the indirect association of this beneficial element in increasing the chlorophyll contents^69^, and consequently, photosynthetic rates^13,76^, which can collaborate with better efficiency in the use of the absorbed Ca.

Results for the shoot and root dry mass corroborate the hypotheses of the Si benefit in mitigating Ca deficiency in quinoa plants. Element supply under Ca deficiency provided better development of stressed plants, attenuating the deleterious effects of nutritional deficiency, and influencing in higher uptake of the missing element, higher green color index, and hence higher chlorophyll content, lower electrolyte leakage, larger leaf area, and higher shoot and root dry mass (Fig. 8b).

### Magnesium (Mg)

At 40 days of cultivation and 10 days of Mg omission, the quinoa plants showed chlorosis symptoms (Fig. 3f). The omission of the nutrient caused a decrease of 91% and 92% in the absence and presence of silicon, respectively, in the accumulation of Mg in comparison to plants submitted to the complete solution (Fig. 4e,f), resulting in deficiency symptoms. Mg deficiency was characterized by standard chlorosis, i.e., between the ribs (Fig. 3i). This occurred because the rib pigment structure remained unchanged for longer periods compared to the structure of chlorophyll in the cells of the leaf limb^76^.

Under Mg deficiency, the Si supply in the solution promoted higher accumulation of Si in the shoots (Fig. 4f), in addition to higher N and K accumulation (Fig. 4a,c). The increased activity of enzymes of the N metabolism^77,78^ resulted in increased N and K accumulation (Fig. 4a,c). The higher K accumulation under Mg deficiency with Si supply was possibly due to the increased H^+^ ATPase activity.

Regarding GCI, regardless of the presence of Si, Mg-deficient plants had lower color intensity than that of plants grown in the complete solution (Fig. 5a). The observed depigmentation was a consequence of the decreased chlorophyll a + b and cx + c contents (Fig. 5b,c). Among the major roles of Mg in plant metabolism, 20% of the Mg present in chloroplast is responsible for the chemical composition of chlorophyll, as it forms the central atom in its molecular structure^41^. Thus, Mg deficiency directly affects the contents of these pigments.

Regardless of the presence of Si, Mg-deficient plants had a significantly higher electrolyte leakage index than that of plants grown in the complete solution (Fig. 6). This may have occurred because a small portion of Mg (5 to 10%), along with Ca, takes part in cell wall synthesis^41^. Thus, the Mg deficiency may have influenced cell wall structure, allowing higher electrolyte leakage.

Regardless of the presence of Si, Mg deficiency resulted in significantly higher F0 than that of plants that were grown in the complete solution. However, in the Mg-deficient plants, the Si supply was not enough to support the photosynthetic metabolism of quinoa plants, which was evidenced by the increased F0 (Fig. 7). Fm was reduced in Mg–deficient plants, and it was significantly differed from that of plants that were grown in the complete solution (Fig. 7B).

For F0/Fm, Mg omission resulted in a significant difference, with higher average F0/Fm in Mg-deficient plants than that in plants grown in the complete solution, highlighting the stress promoted by macronutrient deficiency. Regardless of the Si supply, Mg-deficient plants had a quantum production of 0.23, indicating that the addition of Mg was not enough to attenuate the stress (Fig. 7c).

The Fv/Fm ratio was affected by stress caused by nutrient deficiency. The plants submitted to Mg omission had the Fv/Fm ratio of 0.733, which was lower than the standard average which indicates normal performance of the photosynthetic apparatus (Fig. 7d). Thus, Mg deficiency altered the photosynthetic efficiency of quinoa plants, possibly by damaging the photosynthetic apparatus.

Regardless of the Si supply, Mg-deficient plants had smaller leaf area than that of plants grown in the complete solution, evidencing the importance of this macronutrient for plant development (Fig. 8a). The smaller leaf area may have resulted from the lower pigment content, which led to a lower photosynthetic rate and consequently slower development of quinoa plants.

Despite the Si supply in the nutrient solution of Mg-deficient quinoa plants, the presence of Si was not enough to influence plant development. Thus, the absence of Mg as well as the absence of N were most limiting to crop biomass production. Mg omission led to a decrease of 75% and 79% in shoot dry mass under the absence and presence of Si, respectively, in addition to the 85% decrease in the root system, regardless of the Si supply (Fig. 8B).

### Relationship between treatments and evaluated variables

**Figure 9:**
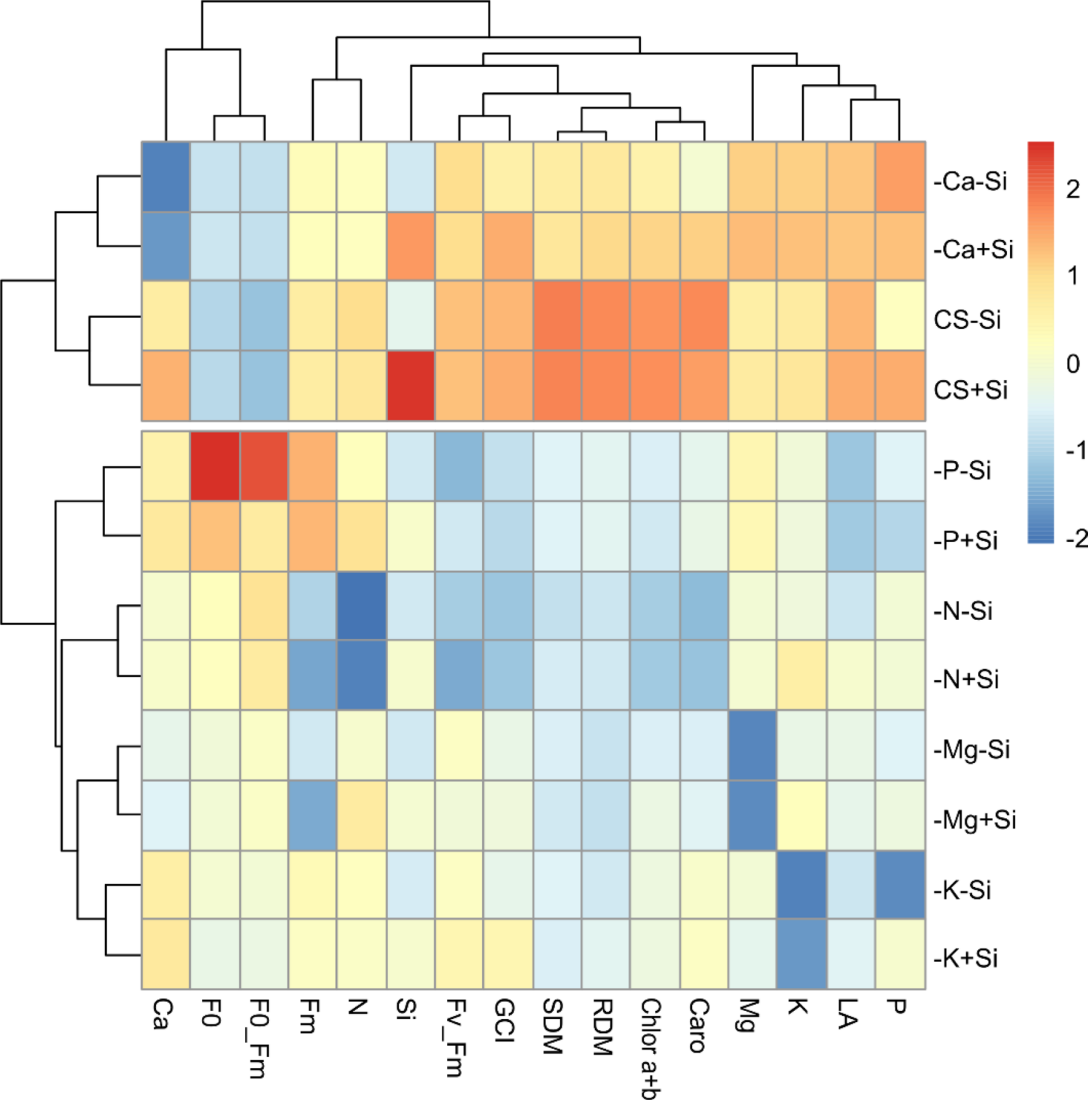
Heatmap built to demonstrate the relationship between treatments and evaluated variables. The package used of R to create this Figure was d3heatmap (v0.4.0, https://cran.r-project.org/web/packages/d3heatmap/index.html).

In order to demonstrate the similarity between the evaluated treatments and the correlation between the variables, a heatmap was constructed (Fig. 9). It is possible to observe the formation of two groups in which the treatments in each group have high similarity for all the evaluated variables. Group I allocated the treatments − Ca − Si, − Ca + Si, CS − Si and CS + Si. The other treatments composed group II. The treatments contained in group I presented the highest means (cells in red) for the variables PSII efficiency (Fv_Fm), green color index (GCI), shoot dry mass (SDM), root dry mass (RDM), total chlorophyll (Chlorophyll a + b), carotenoids (Caro), Mg content, K content, leaf area (LA), and P content. On the other hand, the treatments of group II presented the highest means for initial fluorescence (F0) and basal quantum yield (F0_Fm), which are variables that indicate when the plant is under stress, possibly demonstrating that the treatments in group II were more critical for the development of quinoa plants.

The information obtained by this study allowed us to better understand the Si benefits on the nutritional deficiency of quinoa plants. The findings enable us to safely recommend Si to increase quinoa cultivation sustainability on a global scale due to the wide occurrence of low fertility soils.

## Conclusions

The N, P, K, Ca and Mg deficiencies in quinoa growing caused physiological alterations and visual symptoms characteristic of nutritional deficiency caused by the respective nutrients. The Si supply attenuated the visual effects of the deficiency of all nutrients.

The Si supply under Ca restriction mitigated the deleterious effects of stress by increasing chlorophyll synthesis and protection. For N and Ca, the Si reduced the damages to the cellular wall by the lower electrolyte leakage and consequently increased the dry mass of the quinoa plants.

This study showed important results that are related to the effects of Si in mitigating stress caused by nutritional deficiencies. These results are unprecedented and will serve for future research on the use of Si, minimizing the deleterious effects of nutritional disorders in quinoa plants.
